# Diverse Motor Performances Are Related to Incident Cognitive Impairment in Community-Dwelling Older Adults

**DOI:** 10.3389/fnagi.2021.717139

**Published:** 2021-09-30

**Authors:** Michal Schnaider Beeri, Sue E. Leurgans, David A. Bennett, Lisa L. Barnes, Aron S. Buchman

**Affiliations:** ^1^Department of Psychiatry, Icahn School of Medicine at Mount Sinai, New York, NY, United States; ^2^Sheba Medical Center, The Joseph Sagol Neuroscience Center, Ramat Gan, Israel; ^3^Rush Alzheimer’s Disease Center, Rush University Medical Center, Chicago, IL, United States; ^4^Department of Neurological Sciences, Rush University Medical Center, Chicago, IL, United States; ^5^Department of Psychiatry and Behavioral Science, Rush University Medical Center, Chicago, IL, United States

**Keywords:** aging, cognition, motor function, Alzheimer’s dementia, mild cognitive impairment

## Abstract

**Objective:** Late-life cognitive impairment is heterogeneous. This study examined to what extent varied motor performances are differentially associated with incident Alzheimer’s dementia (AD) and incident mild cognitive impairment (MCI) in older adults.

**Design:** Nested substudy.

**Setting:** Communities across metropolitan Chicago.

**Participants:** African American (*N* = 580) and European American (*N* = 580) adults without dementia, propensity-balanced by age (mean = 73.2; *SD* = 6.0), sex (78.4% women), education (mean = 15.6; *SD* = 3.3) and number of follow ups.

**Measurements:** Cognitive status was assessed annually and based in part on a composite measure of global cognition including 17 cognitive tests. A global motor score was based on 10 motor performances from which 4 motor domains were computed including hand dexterity, hand strength, gait function, and leg strength.

**Results:** During 7 years of follow-up, 166 of 1,160 (14.3%) developed AD. In a proportional hazards model controlling for age, sex, education, and race, each 1-SD higher baseline global motor score was associated with about a 20% reduction in the risk of AD (hazard ratio: 0.81; 95% CI: 0.68, 0.97). Higher baseline motor function was also associated with decreased risk of incident MCI (hazard ratio: 0.79; 95% CI: 0.68, 0.92). Hand dexterity, hand strength and gait function but not leg strength were associated with incident AD and MCI. When including all four motor domains in the same model, results remained the same for incident MCI, while for incident AD, the association with hand strength remained significant.

**Conclusion:** Diverse motor performances are associated with late-life cognitive impairment. Further work is needed to identify specific motor performances that may differentiate adults at risk for future MCI or AD dementia.

## Introduction

Until recently, the defining feature of Alzheimer’s disease (AD) dementia has been progressive cognitive impairment. However, accumulating evidence suggests that AD dementia is a more complex disorder which may affect diverse non-cognitive aging phenotypes including late-life motor impairment (Albers et al., 2015). Poor motor function and a more rapid rate of motor decline are associated with AD pathology (Buchman et al., 2014, 2020), a faster rate of cognitive decline and are independent risk factors for incident MCI and incident AD dementia (Buchman and Bennett, 2011; Mirelman et al., 2014; Ganmore et al., 2020). Yet, these models do not inform about whether motor function occurs before cognitive impairment. In a recent study using novel transition modeling we showed that in many older adults motor impairment may precede and predict the onset of MCI by several years (Buchman et al., 2008; Boyle et al., 2009; Buracchio et al., 2010; Yu et al., 2019a). These data suggest that impaired motor function might serve as an early clinical marker that identifies older adults at risk for developing MCI and AD. If true, this would facilitate early treatments which may prevent the development of cognitive impairment and also improve the homogeneity of adults recruited for clinical dementia trials.

Hand grip strength and gait speed are two commonly employed performances that are simple to measure and have been used in many prior studies to examine the association between motor function and cognition. However, additional work by our group and others has shown that grip strength and gait speed alone is a sensitive but non-specific predictor of incident cognitive impairment (Beeri et al., 2021). Movement is a complex volitional behavior which derives from widely distributed networks that reside in cortical and subcortical brain regions but extends throughout the entire CNS to reach musculoskeletal structures in the periphery. Thus, a more granular assessment of multiple motor performances may be necessary to adequately capture the different facets of motor function which may underlie its association with future cognitive impairment.

Our study seeks to extend prior studies by investigating the role of various motor abilities in predicting incident cognitive impairment We used longitudinal data from a diverse cohort of 1,160 older adults participating in 3 community-based cohort studies, to examine the associations of four easy-to-measure motor factors or abilities (hand strength, hand dexterity, leg strength and gait speed), as well as a composite measure of motor function, with incident MCI and AD dementia.

## Materials and Methods

### Participants

Participants were drawn from three ongoing longitudinal cohort studies of older adults. The Religious Orders Study (ROS) began enrollment in 1993 and the Rush Memory and Aging Project (MAP) began in 1997. Both studies have participants who are mostly European Americans, approximately 6% are African Americans (Bennett et al., 2012, 2018). The Minority Aging Research Study (MARS) is a study of African Americans and began in 2004 (Arvanitakis et al., 2018). All three studies have similar methods of data collection by the same staff including annual clinical visits and cognitive assessments. All studies were approved by the Institutional Review Board of Rush University Medical Center. Written informed consent and an anatomic gift act for brain donation at the time of death was obtained from all study participants in MAP and ROS, and in a subset from MARS.

Eligibility for these analyses required absence of dementia at baseline, with completed cognitive and motor assessments with at least one annual cognitive follow-up evaluation required for longitudinal analyses. At the time of these analyses, 612 African Americans and 1539 European Americans met these criteria Propensity score matching was designed to reduce the confounding of race with other measures, especially since there were racial differences in age and education, both consistently associated with cognition. With the use of propensity scores, we identified 580 African American participants (MARS, 453; MAP, 64; ROS, 63) and 580 European American participants (MAP, 390; ROS, 190) that were balanced in relation to age, sex, education and number of follow-ups which may impact the ability to reliably characterize cognitive trajectories. We used a greedy 5-to-1 digit algorithm in SAS to match propensity scores and identify the subgroups (Rassen et al., 2012). As a result of the propensity balancing, the African American subgroup was similar to the European American subgroup in age at baseline (73.03 vs. 73.37), years of education (15.28 vs. 15.83), and number of annual assessments (7.07 vs. 7.48). The samples also did not differ in sex (78.45% women in both samples).

### Cognitive Assessment and Cognitive Diagnoses

A uniform structured clinical evaluation is performed each year that includes medical history, neurologic examination, and neuropsychological performance tests. Clinical diagnoses were made using a multi-step process, as previously described. Cognitive function testing included 17 performance tests summarized into a composite measure of global cognition as described previously. The cognitive tests data were reviewed by an experienced neuropsychologist who determined if cognitive impairment was present. A clinician, experienced in the evaluation of older persons, reviewed all available data to determine a cognitive diagnosis at each visit using criteria previously described. Dementia was diagnosed using the guidelines of the joint working group of the National Institute of Neurological and Communicative Disorders and Stroke and Alzheimer’s Disease and Related Disorders Association (McKhann et al., 1984). Individuals with cognitive impairment who did not meet dementia criteria were diagnosed with mild cognitive impairment (MCI). Individuals without dementia or MCI were classified as having no cognitive impairment (NCI).

### Motor Assessment

Ten motor performances were assessed. (1, 2) The Jamar hydraulic hand and pinch dynamometers (Lafayette Instruments, Lafayette) were employed to test bilateral grip and pinch strength. (3) Dexterity of the arms was based on the number of pegs placed in the Purdue Pegboard in thirty seconds. Two trials for each hand were averaged to provide a Purdue Pegboard score. (4) An electronic tapper (Western Psychological Services, Los Angeles, CA) was employed to determine how quickly participants were able to tap with their index finger for 10 s. Two trials for each hand were averaged to yield a tapping score. (5–8) We measured the time and number of steps taken to walk eight feet and turn 360° representing gait speed. (9) Participants stood on each leg for 10 s to assess balance. (10) Then they were asked to stand on their toes for 10 s and the time standing was recorded.

These ten measures were scaled and averaged to obtain a summary global motor score as previously described (Fleischman et al., 2015; Buchman et al., 2016). This composite global motor score has been previously reported to be associated with adverse health outcomes (Buchman et al., 2016). In addition, the level and rate of change in global motor score is related to indices of mixed-brain neuropathologies (Buchman et al., 2020). We performed a principal component analysis with varimax rotation to provide empirical support for the notion that the different motor performances tested might capture different facets of motor function. Varimax rotation is commonly used in biomedical and behavioral research since it facilitates interpretation of results by optimizing correlations between variables and factors resulting in high factor loadings for a smaller number of variables and low factor loadings for the rest. In the present study, 10 motor performances were found to cluster in four meaningful yet mostly orthogonal motor abilities (Table 1): (1) *Hand Strength* (grip strength and pinch strength), (2) *Hand Dexterity* (Purdue pegboard placement and finger tapping), (3) *Gait Function* (time to walk eight feet and number of steps), and (4) *Leg Strength and Balance* (standing on one lag and on toes).

**TABLE 1:** Principal component factor analysis* of the 10 performances used to construct the global motor score.

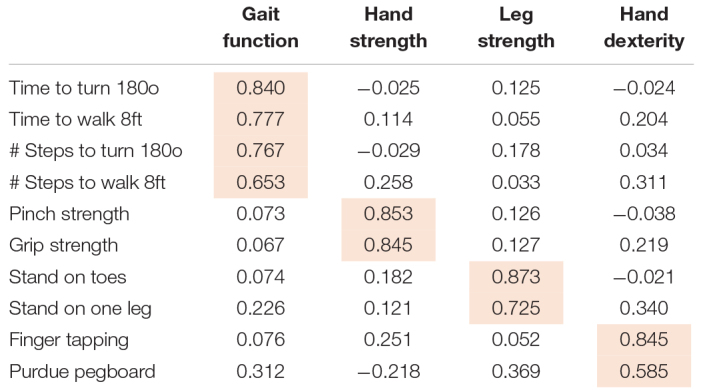

*This table shows the results of principal components analysis for the ten performances used to construct global motor score. Each cell within the four columns shows the factor loading for each of the ten performances after varimax rotation. Highlighted cells show the individual performances whose values were aligned (so that larger values correspond to better performance), scaled and averaged to obtain scores for the four motor domains employed in these analyses.

### Demographics and Other Covariates

Other clinical covariates in these analyses included age, sex years of education and self-reported race obtained at study entry. The sum of three self-reported vascular risk factors including hypertension, type 2 diabetes, and smoking, and the burden of four vascular diseases (heart attack, congestive heart failure, claudication, and stroke), were calculated as previously described (Boyle et al., 2005).

### Statistical Analyses

We employed a set of discrete-time Cox proportional hazards models to examine the association of baseline motor scores (global and specific motor domains) with the first follow-up visit for which a participant received a clinical diagnosis of MCI and AD. These models included terms for demographic variables including age at baseline, sex, education and race. In secondary analyses, we added interaction terms to these models to examine whether these associations varied with demographic factors. Then, we added terms for vascular risk factors and vascular diseases, which may attenuate the association of baseline motor functions and incident cognitive impairment. *A priori* level of statistical significance was 0.05. Models were examined graphically and analytically and assumptions were judged to be adequately met. Programming was done in SAS version 9.3 (SAS Institute Inc., Cary, NC) (SAS Institute, 2012).

## Results

### Metric Properties of Global Motor Score

There were 1,160 participants included in these analyses and their clinical characteristics at baseline are included in Table 2. Global motor scores at baseline ranged from 0.30 to 1.75 (Mean = 1.10; *SD* = 0.21) with higher scores indicating better motor performance. Higher global motor score was associated with younger age (*r* = −0.47, *p* < 0.001), higher education (*r* = 0.23, *p* < 0.001). Motor scores did not differ by sex (*t* = 1.47; *p* = 0.14) or race (*t* = 1.55; *p* = 0.12).

**TABLE 2:** Clinical characteristics at study baseline (*N* = 1,160).

Age, years	73.20 (6.00)
Women,%	78.43
Education, years	15.56 (3.34)
**Race, %**	
African Americans	50%
**Vascular risk factors, %**	
Hypertension	60.09
Diabetes	18.21
Smoking (ever)	39.78
**Vascular disease, %**	
Claudication	5.78
Stroke	6.72
Heart attack	7.51
Congestive heart failure	4.05

### Global Motor Score and Risk of Incident AD

During a mean of about 7.3 years of follow-up (*SD* = 4.0), 166 of 1,160 individuals (14.3%) developed AD. We first examined the relation of baseline global motor score to the risk of incident AD in a Cox proportional hazards model controlling for age, sex, education, and race. In this model, each 1 SD increase in global motor score above the average participant at baseline (female, 73.2 years old with 15.6 years of education) was associated with about a 20% decreased hazard for AD (Table 3, second column). The association of baseline motor function with incident AD did not vary by age, sex, education, or race (all *p*-values for interactions > 0.15). Additional adjustment for cardiovascular risk factors and diseases did not alter the associations of baseline motor function with incident AD (HR: 0.68 (0.54, 0.85); *p* = 0.001). Secondary analyses adjusted for the APOE4 genotype and results remained the same (Supplementary Table 1). Adjustment for baseline cognition (using the Mini Mental State Exam) to examine the unique contribution of motor function beyond cognition led to an attenuation of the association of global motor function with incident AD (Supplementary Table 2).

**TABLE 3:** Associations of global motor function and each individual motor domain with incident AD and incident MCI (HR: 95% CI; *p*-value).

**Motor domain**	**Incident AD**	**Incident MCI**
Global motor function	0.81 (0.68–0.97); 0.025	0.79 (0.68–0.92); 0.002
Hand dexterity	0.85 (0.77–0.95); 0.005	0.86 (0.80–0.93); <0.001
Hand strength	0.92 (0.88–0.97); 0.003	0.93 (0.89; 0.97); <0.001
Gait function	0.92 (0.86–0.99); 0.020	0.91 (0.87–0.96); <0.001
Leg strength	1.00 (0.99–1.00); 0.245	1.00 (0.99–1.00); 0.295

*Each row represents two Cox regression models examining associations of global motor function and each of the individual motor domains with incident AD dementia or incident MCI. Each model includes terms for age, sex, education, and race.*

### Global Motor Score and Risk of Incident Mild Cognitive Impairment

In further analyses we excluded the 247 participants with MCI at study baseline. During a mean of 7.3 years of follow-up (*SD* = 4.03), 335 of 913 participants without cognitive impairment at baseline (36.7%) developed incident MCI.

We first examined the relation of baseline global motor score to the risk of MCI in a Cox proportional hazards model controlling for age, sex, education, and race. In this model, each 1 SD increase in global motor score at baseline was associated with about a 20% decreased risk of MCI (Table 3, third column). The associations of baseline global motor score with incident MCI did not vary by age, sex, education, or race (all *p*-values for interactions > 0.15). Further adjustments for cardiovascular risk factors and diseases did not alter the association of global motor scores and incident MCI [HR: 0.75 (0.64, 0.89); *p* < 0.001]. Secondary analyses adjusting for APOE4 genotype or for baseline MMSE did not alter the associations of motor function with incident MCI (Supplementary Tables 1, 2, respectively).

To examine whether motor function is associated with sub-types of MCI, we repeated the analyses separately for those who developed amnestic MCI (*N* = 123) and non-amnestic MCI (*N* = 122). The association were significant for non-amnestic MCI (HR: 0.91 (0.82, 0.99); *p* = 0.04) and approached significance for amnestic MCI [HR: 0.92 (0.83, 1.01); *p* = 0.08] with similar HRs in both sub-types.

### Motor Abilities and Incident AD Dementia and Incident Mild Cognitive Impairment

**FIGURE 1:**
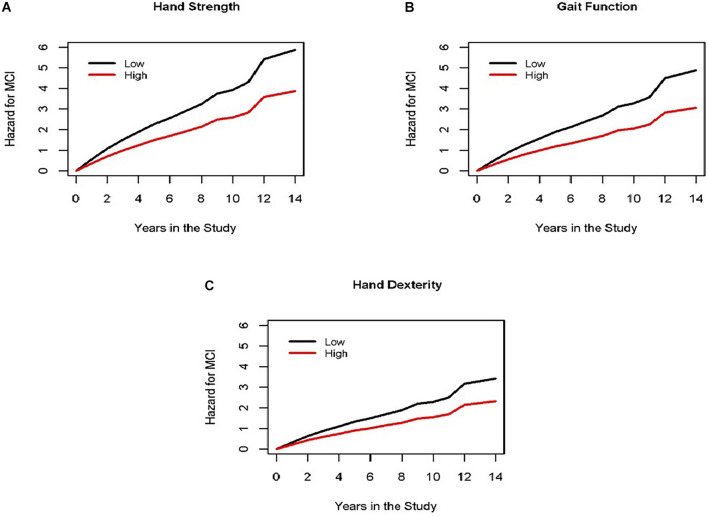
Different motor domains are associated with incident MCI. Each panel (**A** for hand strength, **B** for gait function and **C** for hand dexterity) shows the cumulative hazard of incident MCI during the study for two groups of average female participants, 73 years old, with 16 years of education with high motor function (red line, 90th percentile) vs. low motor function (black line, 10th percentile) at the study baseline for three different domains of motor function (representing results of Model 3 for incident MCI in Table 4).

Global motor score summarized ten motor performances that represent four different motor abilities (Table 1). All four abilities were associated with age and education (see Supplementary Table 3). In further analyses we examined which of the four motor abilities might be driving the associations of global motor score with incident cognitive impairment. As shown in Table 2 and Figure 1, motor dexterity, hand strength and gait speed (but not leg strength) were significantly associated with incident AD and incident MCI, when each of the four motor abilities were analyzed individually. Next we examined which motor abilities were independently associated with incident cognitive outcomes by sequentially adding terms for hand dexterity and leg strength to a model with terms for hand strength and gait speed (Table 4, Models 1–3, upper panel). Hand strength was the only motor component independently associated with incident AD dementia, after adjusting for the other motor abilities (Table 4, Model 3, upper panel). For incident MCI (Table 4, Models 1–3, lower panel), the hand strength, gait speed, and hand dexterity but not leg strength showed independent associations with incident MCI when all four motor abilities were included together (Table 4, Model 3, lower panel).

**TABLE 4:** Associations of individual motor domains driving the associations of global motor score with incident AD and incident MCI (HR: 95% CI; *p*-value).

**Motor domain**	**Model 1**	**Model 2**	**Model 3**
**Incident AD**			
Hand strength	0.93 (0.88–0.99); 0.014	0.94 (0.88–0.99); 0.036	0.94 (0.88–1.00); 0.039
Gait function	0.92 (0.86–0.99); 0.029	0.93 (0.86–1.01); 0.087	0.94 (0.86–1.02); 0.129
Hand dexterity		0.94 (0.82–1.08); 0.394	0.94 (0.82–1.09); 0.430
Leg strength			1.00 (0.99–1.01); 0.870

**Incident MCI**			
Hand strength	0.94 (0.90–0.98); 0.005	0.95 (0.91–0.99); 0.023	0.95 (0.91–0.99); 0.015
Gait function	0.91 (0.87–0.96); 0.001	0.93 (0.88–0.98); 0.008	0.93 (0.87–0.98); 0.011
Hand dexterity		0.92 (0.83–1.01); 0.071	0.90 (0.81–0.99); 0.035
Leg strength			1.00 (1:00–1.01); 0.304

*Each column represents the results of two Cox proportional hazard model showing the associations of different combinations of motor domains identified in the left hand column with incident AD dementia or incident MCI. Each model also includes terms for age, sex, education, and race.*

## Discussion

We examined the association of motor function with the cognitive outcomes for more than 1,100 well-characterized and diverse non-demented older adults who were followed for over 7 years. Better baseline motor function including hand dexterity, hand strength and gait function—was associated with a reduced risk of developing either incident MCI or AD. These findings did not vary with age, sex, education, or race highlighting the robust associations of motor function with incident cognitive impairment in community-dwelling older adults. Additional adjustment for vascular diseases and risk factors also did not alter the results. Further analyses showed that hand strength was the only motor ability independently related to incident AD dementia when adjusting for the other motor abilities. In contrast, hand dexterity, hand strength, and gait function made relatively independent contribution to the association with incident MCI. These findings suggest that testing more diverse motor performances may improve identification of adults at risk for developing incident cognitive impairment.

Prior studies have reported that poor motor function in older adults with normal cognition is associated with future cognitive impairment (Boyle et al., 2009; Mirelman et al., 2014; Yu et al., 2019a; Ganmore et al., 2020). The current study extends prior work in several important ways. First, prior studies have focused on either grip strength or gait speed alone which may not fully capture the diverse facets of motor function, a complex phenotype. In contrast, this study employed a summary measure based on a broader spectrum of upper and lower extremity motor performances which assessed hand strength, hand dexterity, gait function, and leg strength and balance that predicted incident MCI and incident AD dementia. This finding is not surprising since motor function is a volitional behavior controlled by neural control systems that leverage both cognitive and motor resources for the planning, initiation and ongoing execution of all movement (Grafton and Volz, 2019).

Another important aspect of this study is that by examining diverse aspects of motor function, we found that hand strength was independently related to incident AD dementia when adjusting for the other motor domains together in a single model. In contrast, hand dexterity, hand strength, and gait function were independently related with incident MCI. These varied motor performances were easily obtained without the need for wearable sensors or other technology for instrumented testing and can be easily used in varied clinical and community settings. These findings may have important translational consequences as they suggest that employing a more diverse battery of motor performances which can be collected in the community-setting without specialized equipment might provide both clinicians and investigators with a clinical biomarker that can identify adults at risk for distinct cognitive syndromes before the onset of impaired cognition. This has potential to facilitate early and targeted interventions to maintain and forestall cognitive decline in older adults. These data are best viewed as an initial step and highlight the need for further work to identify and optimize the motor performances which improve accuracy of predictions without overburdening staff and those being tested. These efforts must differentiate distinct cognitive phenotypes from other adverse health outcomes which are also related to poor motor function (Buchman et al., 2011).

Our results show that the discrimination, i.e., strength of the association between motor and cognitive status differs between MCI and AD dementia with or without adjustment for cognitive function. These results may derive from the differences in the diagnosis of MCI vs. AD dementia, i.e., there is more random error in the short-term stability of the diagnosis of MCI as compared to AD dementia. Current motor testing focuses almost exclusively on motor execution. While motor function is a volitional behavior, the specific cognitive resources critical to motor planning which precede behavior or the attentional resources that modify movement execution are not part of standard cognitive batteries used to identify dementia or captured by memory complaints. This highlights the importance of further work to clarify the basis for the association of motor function and incident cognitive impairment. If there is a causal relationship between these phenotypes, understanding the biology of poor motor function may shed light on the drivers of both motor and cognitive impairment in older adults and is crucial for choosing motor performances that may increase the discrimination of a clinical motor biomarker for early Alzheimer’s disease and related dementias (ADRD). The associations between motor and cognitive function may not be causal, but rather reflect that both phenotypes share common underlying pathologies. Pathologies of ADRD accumulate outside the cerebrum including brainstem and spinal cord regions, which do not directly affect cognition and might therefore account for impaired motor function preceding cognitive impairment (Yuan et al., 2013; Guo et al., 2016). Finally, decreased motor function may result from other disorders of the central nervous system such as cerebrovascular disease, which we have shown to be associated with both motor (Buchman et al., 2020) and cognitive decline (Yu et al., 2019b). The present study adjusted for cardiovascular risk factors and diseases, which are well-established risk factors for cerebrovascular disease, and the results remained unchanged. Recent work has suggested that conventional self-report history of cerebrovascular disease risk factors and diseases are associated with evidence of cerebrovascular disease pathologies with low discrimination, highlighting the need for more robust vascular risk scores (Oveisgharan et al., 2021).

A limitation of this study is that data are derived from a select cohort of older adults many of whom agreed to brain donation at death, and who may differ in sociodemographic factors, lifestyle, and other exposures from older persons from the general population. Our results show that higher education is associated with better motor function. Education is a complex phenotype and more education may be a proxy for better socioeconomic status and access to health care, especially in old age (Fleischman et al., 2015), and may provide motor reserve (Elbaz et al., 2013).” While our sample was diverse and included large numbers of African Americans and European Americans, it will be important to replicate our findings in additional cohort studies. Strengths of our study include the structured and validated cognitive measures and motor function assessment that encompasses a broad range of common motor performances, in a large diverse sample of older adults. The study used propensity score matching, ensuring that the two racial groups are balanced on important variables that otherwise may have confounded the results, especially age and education, for which there were racial differences. It is possible that this strict balancing diminished the strength of the associations between motor function and incident MCI and AD. It is noteworthy, that ultimately, to assess cognitive decline, cognitive assessments must be applied. However, we have shown that motor function impairment develops significantly earlier than cognitive impairment. Therefore, assessment of these easy to measure motor performances, may provide important information about risk of future cognitive decline, and allow for early intervention. The distinct motor performances were empirically defined by principal component analyses and are thematically sound; they can be easily obtained in diverse clinical settings as they do not require specialized sensors, potentially facilitating prediction of older adults at risk of future cognitive impairment outside of the research setting.

## Data Availability Statement

The raw data supporting the conclusions of this article will be made available by the authors, without undue reservation.

## Ethics Statement

The studies involving human participants were reviewed and approved by the Institutional Review Board of Rush University. The patients/participants provided their written informed consent to participate in this study.

## Author Contributions

MB analyzed the data, interpreted the results, and wrote the manuscript. SL analyzed the data and reviewed the manuscript. DB developed the design, collected the data, and reviewed the manuscript. LB collected the data and reviewed the manuscript. AB developed the design, collected the data, and supervised the manuscript write up. All authors contributed to the article and approved the submitted version.

## Conflict of Interest

The authors declare that the research was conducted in the absence of any commercial or financial relationships that could be construed as a potential conflict of interest.

## Publisher’s Note

All claims expressed in this article are solely those of the authors and do not necessarily represent those of their affiliated organizations, or those of the publisher, the editors and the reviewers. Any product that may be evaluated in this article, or claim that may be made by its manufacturer, is not guaranteed or endorsed by the publisher.
