# Supplementary material for: Diverse Motor Performances Are Related to Incident Cognitive Impairment in Community-Dwelling Older Adults
Source: Front Aging Neurosci. 2021 Sep 30;13:717139. doi: 10.3389/fnagi.2021.717139 (PMC8514826; doi:10.3389/fnagi.2021.717139)
Supplement: Supplementary file 1 [file Data_Sheet_1.pdf]

Electronic Supplement

A battery of 21 cognitive performance tests was administered in an approximately 1-hour testing session. One test, the Mini-Mental State Examination, was used to describe the cohort. A composite measure of global cognition was made by averaging the z-scores of all other tests. Since neuropsychological tests do not measure cognition uniformly across different levels of education, an algorithm rated impairment in 5 cognitive domains (i.e., orientation, attention, memory, language, perception) based on educationally adjusted cutoff scores on 11 cognitive tests has been developed[1-3]. After reviewing all cognitive data, education, occupation, and ratings of sensory problems, motor problems, and the participant's level of effort, a neuropsychologist agreed or disagreed with the algorithmic rating of each cognitive domain. In the event of disagreement, the neuropsychologist supplied a new impairment rating. Dementia required meaningful decline in cognitive function with impairment in multiple areas of cognition, and AD required dementia and progressive loss of episodic memory based on the criteria of the joint working group of the National Institute of Neurologic and Communicative Disorders and Stroke and the AD and Related Disorders Association (NINCDSADRDA). The diagnosis of MCI required cognitive impairment in the opinion of a neuropsychologist in the absence of dementia in the opinion of the clinician. MCI was classified as amnestic if the memory domain was impaired and non-amnestic if memory was not impaired, as previously reported[4, 5]. These MCI criteria have been related to intermediate levels of cognitive decline[4, 6], and Alzheimer's disease pathology compared to dementia and no cognitive impairment.

Supplemental Table 1. Associations of global motor function and each individual motor domain with incident AD and incident MCI (HR: 95% CI; p-value)

Motor Domain	Incident AD	Incident MCI
**Global Motor Function**	0.90 (0.83, 0.98); **0.012**	0.87 (0.82, 0.93); **<0.001**
**Hand Dexterity**	0.91 (0.85, 0.97); **0.002**	0.92 (0.88, 0.96); **<0.001**
**Hand Strength**	0.92 (0.88-0.97); **0.003**	0.93 (0.89; 0.97); **<0.001**
**Gait Function**	0.92 (0.86, 0.99); **0.023**	0.91 (0.87, 0.96); **<0.001**
**Leg Strength**	1.00 (0.99, 1.00); 0.44	1.00 (0.99, 1.00); 0.22

Each row represents two Cox regression models examining associations of global motor function and each of the individual motor domains with incident AD dementia or incident MCI. Each model includes terms for age, sex, education, race and APOE4 genotype.

Supplementary Table 2. Associations of global motor function and each individual motor domain with incident AD and incident MCI (HR: 95% CI; p-value)

Motor Domain	Incident AD	Incident MCI
**Global Motor Function**	0.93 (0.86, 1.01); 0.089	0.89 (0.84, 0.95); **<0.001**
**Hand Dexterity**	0.90 (0.80, 1.01); 0.061	0.89 (0.82, 0.96); **0.004**
**Hand Strength**	**0.923 (0.88, 0.99); 0.016**	0.93 (0.89, 0.97); **0.001**
**Gait Function**	0.95 (0.89, 1.02); 0.138	0.92 (0.88, 0.96);**<0.001**
**Leg Strength**	1.00 (0.99, 1.00); 0.72	1.00 (0.99, 1.00); 0.56

Each row represents two Cox regression models examining associations of global motor function and each of the individual motor domains with incident AD dementia or incident MCI. Each model includes terms for age, sex, education, race and baseline Mini Mental State Exam.

**Supplemental Table 3- Pearson correlations of motor domains with age and education** (r, p-value)		
**Motor domain/performance**	**Age**	**Education**
**Motor dexterity**	-0.39, <0.001	0.24, <0.001
**Hand strength**	-0.35, <0.001	0.14, <0.001
**Gait function**	-0.30, <0.001	0.13, <0.001
**Leg strength**	-0.37, <0.001	0.19, <0.001

1. Bennett DA, Wilson RS, Schneider JA, Evans DA, Beckett LA, Aggarwal NT, Barnes LL, Fox JH, Bach J. Natural history of mild cognitive impairment in older persons. *Neurology.* 2002;59(2):198-205.

2. Bennett DA, Schneider JA, Aggarwal NT, Arvanitakis Z, Shah RC, Kelly JF, Fox JH, Cochran EJ, Arends D, Treinkman AD, Wilson RS. Decision rules guiding the clinical diagnosis of Alzheimer's disease in two community-based cohort studies compared to standard practice in a clinic-based cohort study. *Neuroepidemiology.* 2006;27(3):169-176.

3. Bennett DA, Schneider JA, Buchman AS, Mendes de Leon C, Bienias JL, Wilson RS. The Rush Memory and Aging Project: study design and baseline characteristics of the study cohort. *Neuroepidemiology.* 2005;25(4):163-175.

4. Wilson RS, Leurgans SE, Boyle PA, Bennett DA. Cognitive decline in prodromal Alzheimer disease and mild cognitive impairment. *Arch Neurol.* 2011;68(3):351-356.

5. Wilson RS, Schneider JA, Arnold SE, Tang Y, Boyle PA, Bennett DA. Olfactory identification and incidence of mild cognitive impairment in older age. *Arch Gen Psychiatry.* 2007;64(7):802-808.

6. Boyle PA, Wilson RS, Aggarwal NT, Tang Y, Bennett DA. Mild cognitive impairment: risk of Alzheimer disease and rate of cognitive decline. *Neurology.* 2006;67(3):441-445.
